# Risk factors influencing swine influenza A virus infection in South Korea: A systematic review and meta-analysis of prevalence and seroprevalence

**DOI:** 10.3389/fvets.2022.1003351

**Published:** 2022-09-29

**Authors:** Simin Lee, Eurade Ntakiyisumba, Jae-Won Seol, Gayeon Won

**Affiliations:** College of Veterinary Medicine, Jeonbuk National University, Iksan, South Korea

**Keywords:** swine influenza A virus, pigs, prevalence, seroprevalence, systematic review, meta-analysis

## Abstract

The past and current burden of swine influenza A viruses (swIAV) must be estimated since pigs act as mixing vessels and are considered a potential source of newly emerging IAV variants. The objective of this systematic review and meta-analysis was to integrate data on the prevalence and seroprevalence of swIAV in South Korean domestic pigs and evaluate important risk factors that influence these outcomes. Eight databases were searched for studies that evaluated the prevalence and seroprevalence of swIAV in South Korean pigs using a specified search string; twenty-seven eligible studies were identified after application of a set of pre-determined inclusion criteria by three authors. The reported prevalence and seroprevalence were pooled separately in proportions between 0 and 1, using a random-effect meta-analysis. To identify and quantify potential sources of heterogeneity, subgroup, and meta-regression analyses were conducted using covariates (publication type, swIAV subtype, growth stage of pigs, sampling region, publication year, sampling season, facility, detection method, sample type, and sample size). The overall prevalence and seroprevalence in domestic pigs were 0.05 [95% confidence intervals (CIs): 0.05–0.12] and 0.35 (95% CIs: 0.14–0.63), respectively. To identify the impact of covariates on effect size, a suitable meta-regression model was determined using predictor importance estimates with corrected Akaike information criterion values. Consequently, the best-fit model included two covariates, publication year and sample size, which were significantly associated with high heterogeneity in the subgroup analysis. Furthermore, data visualization depicted a significant non-linear association between swIAV prevalence and seroprevalence and specific growth stages of pigs. These findings suggest that the periodic monitoring of pigs at different growth stages in large farms may help to establish the status of swIAV-spread across species in the region, and thereby minimize pandemic risk.

## Introduction

Swine influenza (SI), a highly infectious viral disease, has a significant impact on the swine industry and human health worldwide. Pigs are considered mixing vessels for swine influenza A virus (swIAV) because the respiratory epithelial cells of pigs have receptors to which the influenza virus of human, swine, and avian origin can adhere (1–3). Triple-reassortant swIAVs isolated from pigs in the United States in 1998 contained genes derived from human, swine, and avian IAVs, suggesting cross-species transmission (4). Since 2005, swIAV infections have been reported in humans who come in contact with pigs, and the symptoms range from headache without fever to diarrhea (4). Consequently, the 2009 H1N1 pandemic (H1N1pdm09) virus, derived from reassortment between the North American and Eurasian viral lineages, triggered a pandemic crisis that spread worldwide through pig-to-human and human-to-human transmission between March 2009 and August 2010 (1, 5). In South Korea, more than 750,000 cases of H1N1pdm09 infection were confirmed during this period, with a case fatality rate of approximately 0.03% (6). However, we still do not have a clear understanding of the risk of emerging new recombinant swIAVs and their source.

Continuous active surveillance is essential to anticipate the probability of the occurrence of novel recombinant swIAVs and establish preventative countermeasures (7). Active surveillance is focused on pig herds with risk factors, as continuous active surveillance of all pigs nationwide would be labor- and resource-intensive. Some studies have reported risk factors for swIAV, but these are inconsistent because of the varied ecosystems, animal husbandry systems, and different populations of the investigated herds in individual studies (8). According to research carried out in UK, indoor rearing of pigs, high density of pigs per water space, and the sampling season are possible risk factors for swIAV infection (9). Pig farms in France are at risk for swIAV seropositivity because of the number of other pig herds within 2 km, high stocking density and low temperatures in breeding facilities, and the absence of an all-in/all-out system (10). A Spanish study identified the existence of open walls across pens, increased replacement rates in pregnancy units, and unrestricted access to farms as risk factors for swIAV seropositivity (11). Farm size, presence of other animals, breeding period of sows, existence of a poultry farm within 1 km, and purchasing pigs from pig collectors have been documented as risk factors for swIAV transmission in pig farms in Malaysia (12) and Indonesia (13). Therefore, risk factors in different countries must be evaluated based on the environment and breeding management systems followed in each.

By comprehensively integrating the results of previous studies, meta-analysis can expand the sample size and statistical power to further improve the effect size estimate, and examine variability between studies (14). A systematic review can synthesize the scientific evidence obtained through a meta-analysis and summarize the evidence for risk factors (15). Therefore, systematic reviews and meta-analyses of prior research could provide deeper insights by collecting and summarizing information on the prevalence, seroprevalence, and risk factors of swIAV in domestic pigs. Despite this, to our knowledge, no systematic reviews and meta-analyses on the risk factors for swIAV in South Korea have been undertaken to date. Against this background, our goal was to investigate the transmission of swine influenza in Korean swine farms through a systematic review and meta-analysis using previously reported data on the prevalence and seroprevalence of swIAV in South Korea. Furthermore, we hypothesized that the prevalence and seroprevalence of swIAV in the domestic pig population depended on study- and trial-level characteristics and attempted to identify the risk factors that might influence the dependent variable. This study may provide information necessary to establish a more effective swIAV-monitoring method and suitable countermeasures to prevent cross-species transmission, which may be the potential pre-pandemic in the future.

## Materials and methods

### Study protocol and eligibility criteria

The procedure for this systematic review and meta-analysis was developed following the PRISMA-P guidelines (Supplementary Tables 1, 2) (16). Table 1 lists the components of the population, exposure, control, outcomes, and study (PECOS) that were included to assess the eligibility of the primary studies identified in the search.

**Table 1:** Eligibility criteria and search strings for investigating the prevalence or the seroprevalence of swine influenza A virus (swIAV) for domestic pigs in South Korea.

	**Inclusion criteria**	**Exclusion criteria**
Population	• Domestic pigs in South Korea	• Other animals except pigs • Other country • Wild animals
Outcomes	• Prevalence of swine Influenza A virus • Seroprevalence of antibodies of swine Influenza A virus • Isolation rate of swine Influenza A virus	• Experimental results from isolated virus • Genomic analysis
Study type	• Cross-sectional studies	• Ecological studies • Descriptive observational studies • Case-control studies • Cohort studies Reviews
Search strings		

(porcine OR swine OR pig or pigs OR piglet OR piglets OR gilt OR gilts OR sow OR sows OR hog OR hogs OR weaner OR finisher OR finishers) AND (Korea*) AND (avian influenza* OR swine influenza OR AI OR bird flu OR avian flu OR influenza A virus) AND (prevalence OR inciden* OR proportion OR cases OR surveillance OR seroprevalence).

Population (P): The population of interest for determining swIAV transmission in South Korea was defined as domestic pigs.

Exposure of interests (E) and control (C): As there was no control group in the prevalence and seroprevalence studies, these two categories were not defined in this study.

Outcomes (O): The prevalence of swIAV and the seroprevalence of antibodies against swIAV were assessed. Reverse transcription polymerase chain reaction (RT-PCR) is mainly used to screen for the presence of swIAV, but it is difficult to confirm the integrity of the virus because it detects target gene fragments (17). Therefore, the isolation rate, which can confirm the integrity of the virus, was also deemed a suitable outcome for this study.

Study design (S): This review included cross-sectional studies to assess the prevalence and seroprevalence of swIAV. Ecologic, descriptive observational, case-control, and cohort studies were not suitable for this review.

### Search strategy and study selection

Relevant primary studies were identified in the online databases using search strings comprising four keywords: swine (population), South Korea (population), swIAV (outcomes), and prevalence or seroprevalence (outcomes) (Table 1). The following databases were searched for relevant studies that were published until December 2021, in all languages: PubMed, Scopus, Web of Science, Research Information Sharing Service, ScienceOn, and DBPIA. The following databases were also searched for relevant gray literature: Google Scholar, Proquest Dissertations, and Thesis. The full text, which is difficult to access, was requested from the Foreign Research Information Center at Jeonbuk National University (http://www.fric.kr/user/centerMainView.do?centerId=jbnu). The search strings used to identify eligible studies were customized for each database, considering the differences in their indexing or functionality. EndNote X9 (Clarivate, Philadelphia, PA, USA) was used to import the search results and duplicates were omitted. Two independent researchers determined study eligibility using a two-level screening. The first screening consisted of titles and abstracts, and the final screening comprised the full texts. Disagreements were resolved by consensus or arbitration by an independent reviewer.

### Data extraction

Prevalence and seroprevalence data were extracted separately from eligible studies and organized into Excel files. The following data were extracted: author's name, number of tested samples, and number of positive samples or isolated viruses. The following covariates were selected in advance for use in subgroup and meta-regression analyses: publication type (published and not published); swIAV subtype; growth stage of pigs (farrowing, post-weaning, growing, finishing, gilt, and sow); sampling region (Gyeonggi, Gangwon, Chungcheong, Jeolla, Gyeongsang, and Jeju); publication year; sampling season (first and second halves); facility (farm and slaughterhouse); detection method; sample type; and sample size. In the case of sampling on farms, additional information on farms was also reviewed (herd size, pig density, and accommodation types) (9). If the variables were not found in the literature, they were marked as “not reported.” An adequate sample size to estimate the pooled prevalence and seroprevalence was calculated to achieve the desired precision of 95% confidence intervals (CIs) using previously reported data and methodology (at least 600 and 750 samples were required for prevalence and seroprevalence, respectively) (18–20). Attempts were made to contact the authors for further information related to their study if necessary. If the authors did not respond, the issue was resolved by consensus among the researchers.

### Risk-of-bias assessment

Three researchers independently assessed the quality of the primary studies selected for meta-analysis using the JBI critical appraisal checklist for prevalence studies (21). Each study was rated as having a low, high, or unclear risk of bias for eight of the nine questions included in the checklist, with the exception of one question regarding the survey. A final decision was reached through consensus among the researchers and any disagreements were resolved through the arbitration of an independent reviewer.

### Meta-analysis

The meta-analysis was conducted with the “meta,” “metafor,” “dmetar,” and “ggcorrplot” packages in the software R version 4.1.2 (R Studio version 1.4) (15, 22–26). Random effects models with 95% CIs were used to analyze the data, considering the variation between the studies. The proportion data of both prevalence and seroprevalence were pooled by applying a generalized linear mixed model (GLMM) with logit transformation (27, 28). The heterogeneity variance τ^2^ estimates were evaluated by using the maximum likelihood estimator. Subsequently, the 95% CIs were calculated using the Knapp-Hartung adjustment considering the uncertainty of the estimates caused by the heterogeneity (29). The results of analyses were represented as forest plots with 95% CIs. For the final presentation, the parameter estimates ranging from –∞ to ∞ of the logit-transformed proportion were converted into conventional proportions ranging from 0 to 1 using the inverse logit transformation. Between-study heterogeneity was examined based on the Cochran's *Q*-test, τ^2^ estimates, and *I*^2^-values (30). If findings showed significant heterogeneity (*I*^2^ > 50%), the potential patterns and sources of heterogeneity were explored through subgroup analysis using the aforementioned covariates. Subgroup analysis was only performed when covariate data were reported from at least five studies (31). As seroprevalence data have been detected only in serum samples collected from the target farms, a subgroup analysis of seroprevalence based on facility type (farm or abattoir) or sample type was not performed. The subgroup analysis based on farm details (herd size, pig density, and accommodation types) was also not conducted as the relevant data were reported in only four eligible studies.

### Meta-regression analysis and data visualization

The subgroup analysis guided the selection of covariates for inclusion in the meta-regression model, which was developed using a combinatorial approach (15, 32). Multicollinearity was measured between covariates to verify whether the regression assumption was satisfied (33). Pairs of covariates were revisited if the correlation coefficient between two covariates was >0.7 or lower than −0.7, and then one covariate-of-interest was included in the analysis (15, 33). Subsequently, to quantify the impact of study-level covariates in the regression model, estimates of predictor importance were established (15). The estimates showed the relative importance of each selected predictor variable across all linear regression models. Variables with values close to 1 were estimated to have largely contributed to the final regression model (15). To identify a suitable and best-fit model, the corrected Akaike information criterion (AICc) was used, which accurately estimates the prediction error in the statistical model without being influenced by a small sample size (15). Furthermore, potential regressions such as quadratic or cubic splines between predictor variables and effect size may not be considered in this linear meta-regression analysis. Thus, the relationship between covariates, which were not selected in the final regression model but considered significant in the subgroup analysis, and the effect size were visualized using weighted scatter plots. Publication bias was quantified using contour-enhanced funnel plots and Egger's regression test (34, 35).

## Results

### Study selection

**Figure 1:**
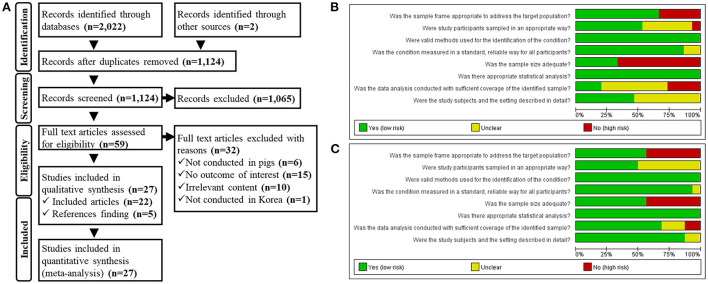
**(A)** Preferred Reporting Items for Systematic Review and Meta-Analysis Protocols (PRISMA-P) flowchart. Quality assessment of the eligible studies including the data for **(B)** swine influenza A virus (swIAV) prevalence and **(C)** seroprevalence of swIAV in South Korean domestic pigs.

We identified 2,024 documents from the database and gray literature searches. After eliminating duplicates, 1,124 electronic records remained. A further 1,065 documents were eliminated after title/abstract eligibility screening, and another 32 documents were removed because of ineligible populations, outcomes, countries, and irrelevant content during full-text screening. After screening, 22 documents were deemed eligible for inclusion in the study. Five additional records were identified by reference-list screening, resulting in 27 documents (Figure 1A).

### Study characteristics and outcomes

Supplementary Tables 3, 4 summarize the features of the 27 studies (36–62). Briefly, 11 studies (37–39, 44, 47–50, 55, 60, 61) reported the prevalence of swIAV solely, and 12 studies (36, 40–42, 45, 46, 51–53, 56, 58, 59) reported the seroprevalence of swIAV antibodies. Four studies (43, 54, 57, 62) reported both the prevalence and seroprevalence of swIAV. The eligible studies comprised 24 published studies (36–59), two dissertations (60, 61), and one government report (62). The prevalence of swIAV has been reported in farms (37–39, 43, 44, 47, 49, 54, 60–62) and abattoirs (48, 50, 55, 57), whereas the seroprevalence of swIAV antibodies has been reported exclusively in farms (36, 40–43, 45, 46, 51–54, 56–59, 62). Among the seroprevalence studies, only five studies (36, 41, 45, 46, 58) reported farm details. RT-PCR and real-time RT-PCR (rRT-PCR) were employed to detect swIAV antigens in 12 studies (37–39, 44, 48–50, 54, 56, 57, 60, 62), whereas the hemagglutination (HA) assay and immunohistochemistry (IHC) staining were employed in two (47, 61) and one (43) studies, respectively. Nasal swabs (38, 39, 47, 49, 54, 61, 62) and lung tissues (37, 39, 43, 44, 48–50, 54, 55, 57) were used as samples for prevalence testing, and feces was used in only one study (60). In 11 studies (42, 43, 45, 51–54, 56–58, 62), hemagglutination-inhibition (HI) assay was used to detect swIAV antibodies, whereas ELISA was used in five studies (36, 40, 41, 46, 59). The sampling season was reported in eight studies (38, 40, 41, 50, 51, 55, 57, 59), and growth stages (36–38, 40–46, 48, 50, 53, 55, 57, 59), swIAV subtype (36–38, 40–42, 44–47, 49–59, 61, 62), and sampling provinces (36–42, 44–47, 49–62) were reported in most studies. In previous studies (39, 43, 48, 60) that detected antigens targeting the *M1* gene, which is common to all subtypes of swIAVs (63), the subtypes could not be distinguished.

### Risk of bias within studies

The results of the quality assessment of eligible studies that reported the prevalence and seroprevalence of swIAVs in domestic pigs are shown in Figures 1B,C, respectively. Studies reporting both prevalence and seroprevalence evaluated the risk of bias by classifying each outcome. The sample frame was selected adequately for prevalence studies in which samples were collected from the entire country (38, 39, 47, 60, 62) or a specific province (48, 50, 55, 57, 61), as samples were extracted from within the relevant regions to determine the prevalence therein. As the specimens that were referred to diagnostic centers were mainly collected from individuals with clinical symptoms (37, 43, 44, 49, 54), it was difficult to assume that the sample frame was the general population of pigs; however these prevalence studies applied an appropriate sampling method called census, which examined all samples submitted within an established sample frame. The prevalence studies sampled from random individuals (47, 50, 57) were judged to have a low bias toward the sampling method; however, one study (38), wherein samples were collected only from individuals with clinical symptoms, was judged to have a high bias in the sampling method. Samples in all studies had been analyzed using appropriate methods, including RT-PCR (37–39, 44, 48, 49, 55, 57, 60, 62), rRT-PCR (50, 54), HA assay (47, 61), or IHC staining (43). In all studies, except two (61, 62), the results of experimental methods had read with distinguishing criteria. Prevalence studies with small (37–39, 43, 44, 48–50, 57, 61) and large sample numbers (47, 54, 55, 60, 62) were distinguished according to adequate sample size. All studies explicitly stated the number of tested samples and the number of positive cases or viruses detected. Prevalence studies that contained divided subgroups according to sampling seasons (50, 55, 60), sampling year (37, 54, 55, 60, 62), and province (37, 47, 60, 62) were judged to allow the comparison of the study sample with the population of interest. Prevalence studies with a similar number of samples for each distinct subgroup (50, 54, 55) were judged to have low coverage bias. Otherwise, coverage bias was evaluated as high (37, 47, 60, 62).

Seroprevalence studies, in which samples were collected from the whole country (42, 45, 46, 52, 53, 56, 62) or a specific province (36, 57), were judged to have an adequate sample frame. Seroprevalence studies using samples that had been referred to diagnostic centers (40, 41, 43, 51, 54, 58, 59) used an inadequate sample frame. Seroprevalence studies that sampled either the total inspection within the sampling frame (51, 54) or random individuals (36, 40, 41, 45, 46, 59) were judged to have a low bias toward the sampling method. In all experiments, antibodies were detected using appropriate methods, including the HI assay (42, 43, 45, 51–54, 56–58, 62) and ELISA (36, 40, 41, 46, 59). In all studies, except one (62), the results of experimental methods had read with distinguishing criteria. Seroprevalence studies with small (36, 41–43, 45, 46, 56) and large sample numbers (40, 51–54, 57–59, 62) were distinguished according to adequate sample size. All studies explicitly stated the number of tested samples and positive cases. Seroprevalence studies that divided subgroups into sampling seasons (40, 41, 51, 52, 57–59), sampling year (36, 52, 54), growth stage (36, 40–43, 59), and province (46, 53, 58, 62) were judged to allow comparison of the study sample with the population of interest. Seroprevalence studies with a similar number of samples for each distinct subgroup (36, 40, 41, 43, 51, 53, 54, 57–59, 62) were considered to have low coverage bias. Otherwise, the coverage bias was high (42, 46).

### Results of the meta-analysis

#### Forest plots and subgroup analysis

**Figure 2:**
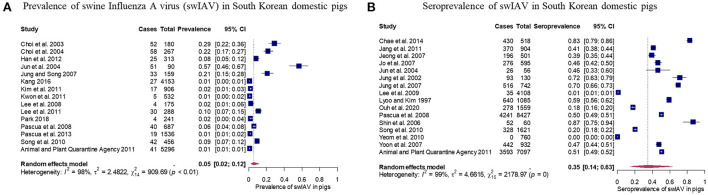
Results of meta-analysis of prevalence and seroprevalence of swine influenza A virus (swIAV) in domestic pigs. **(A)** Forest plot of prevalence with 95% confidence interval (CIs) of swIAV in domestic pigs. **(B)** Forest plot of seroprevalence with 95% CIs of swIAV in domestic pigs. Size of the blue square corresponds to the weight of each study. Vertical dotted line symbolizes the overall effect size.

Fifteen studies evaluated the prevalence of swIAV in domestic pigs in South Korea. The pooled prevalence was 0.05 with 95% CIs of 0.02–0.12 (Figure 2A). The pooled prevalence indicated that approximately 2–12% of the domestic pigs in South Korea have swIAV antigens in their bodies. Studies evaluating prevalence of swIAV showed significant heterogeneity between studies (τ^2^ = 2.48; *p*-value of Q-test < 0.0001; *I*^2^ value = 98.5% [98.1%; 98.8%]). Sixteen selected studies investigated the seroprevalence of swIAV in domestic pigs in South Korea. The pooled seroprevalence was 0.35 with 95% CIs of 0.14–0.63 (Figure 2B). The pooled seroprevalence suggests that 14–63% of the domestic pigs in South Korea have had swIAV antibodies in their body. Studies evaluating seroprevalence of swIAV showed significant heterogeneity between studies (τ^2^ = 4.66; *p*-value of Q-test = 0; *I*^2^ value = 99.3% [99.2%; 99.4%]). Subgroup analyses were conducted to account for the high between-study heterogeneity of the pooled prevalence and seroprevalence. The results of the analyses are summarized in Supplementary Tables 5, 6. Briefly, the following characteristics were significantly associated with variation in the pooled prevalence (*P* < 0.001): publication type, sample size, sample type, detection methods, growth stage, and publication year. The subgroups, sample size (*P* < 0.05) and growth stage (*P* < 0.0001), revealed the potential source of heterogeneity within the pooled seroprevalence data. The impact of these covariates was further assessed by the meta-regression analysis.

#### Meta-regression model selection and data visualization

**Figure 3:**
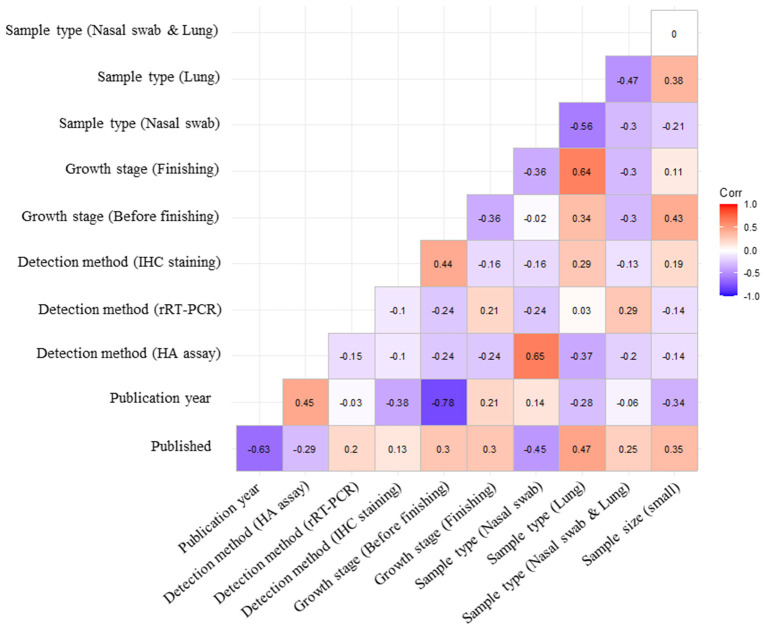
Assessment of the degree of association between the covariates. Each subcategory of covariates is listed on both the x- and y-axes. Spearman's rank correlation coefficient (ρ) was computed to evaluate multicollinearity among each covariate.

**Figure 4:**
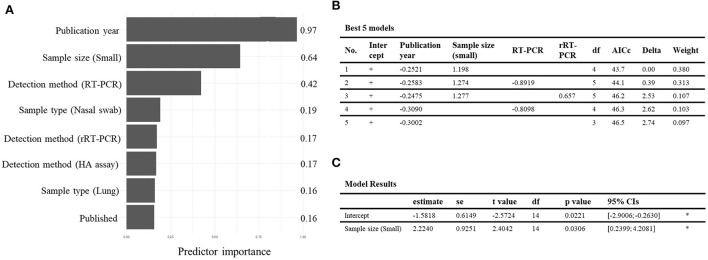
Results of meta-regression analysis of the prevalence and the seroprevalence of swine influenza A virus (swIAV) in domestic pigs. **(A)** Model-averaged predictor importance plot for multiple meta-regression model of the swIAV prevalence data. **(B)** The best five multiple meta-regression models with the lowest corrected Akaike's information criterion (AICc) for the swIAV prevalence outcome. **(C)** Meta-regression model with sample size for the swIAV seroprevalence outcome.


The careful selection of covariates that must be included into a meta-regression model is critical for obtaining validated results. Multiple meta-regression analysis was implemented to explore associations between study-level covariates (publication type, publication year, tested sample size, sample type, detection method, and growth stage) and pooled prevalence (32). Covariates with a *p*-value < 0.05 in the subgroup analysis were entered into the regression model. In the correlation matrix, a high negative correlation between the publication year and growth stage of pigs (*r* = −0.78) was detected (Figure 3). Owing to multicollinearity, only the publication year was included in the model. A suitable regression model was selected using the model-averaged prediction importance plot and by comparing the AICc values of a list of the five best-fit models (Figure 4). The plot shows that the publication year and sample size had relatively high value among the other candidate variables (Figure 4A). Among the examined models, both publication year and sample size were included in the top three models with the lowest AICc values (Figure 4B). To minimize the risk of overfitting, a model with a lower number of terms is considered the best. The final model was:

$$\begin{array}{l} logit(Y_{prevalence})=1-0.2521\times X_{publicationyear}\\ \;+1.198\times X_{smallsamplesize} \end{array}$$

where the term *X*_*publicationyear*_ is the continuous variable denoting the publication year, and the term *X*_*smallsamplesize*_ is the binary variable stratified by the sample size (i.e., 1 for a small sample size <600 and 0 for a large sample size >600). The term *logit*(*Y*_*prevalence*_) is the logit-transformed value of prevalence. For the seroprevalence data, a simple meta-regression analysis was conducted using only the sample size, which was significantly associated with heterogeneity in the subgroup analysis; the growth stage, another significant variable, was not the study-level covariate (Supplementary Table 4). The result indicated that the logit value of seroprevalence significantly increased by 2.2240 when a small sample size of <750 was applied (*P* < 0.05) (Figure 4C).

**Figure 5:**
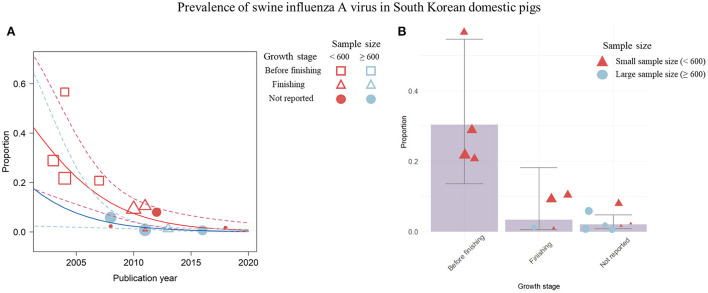
Visualization of the prevalence data for swine influenza A virus (swIAV) by the significant covariates; publication year, sample size, and growth stage of pigs. **(A)** Change in swIAV prevalence according to the publication year visualized by a weighted scatter plot. Solid red line denotes the regression describing association among the prevalence, the small sample size, and the publication year. Solid blue line indicates regression depicting the relationship among the prevalence, the large sample size, and the publication year. Dashed lines show 95% CIs of the regression model. **(B)** swIAV seroprevalence proportion by each growth stage using the bar plot. Circles or triangles indicate individual studies and the size of the figures corresponds to the weighted average of effect size of each study. The sample size is represented by red (small) and blue (large) colors. Error bar represents 95% CIs.

**Figure 6:**
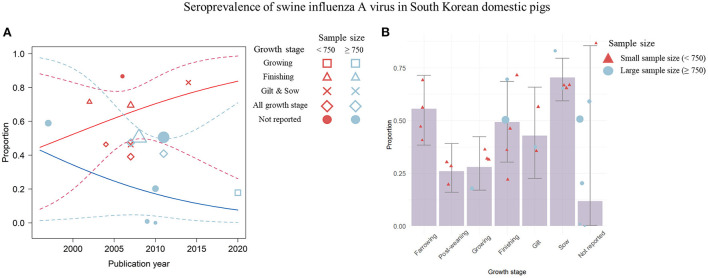
Visualization of the seroprevalence data determined by the significant covariates; the publication year, the sample size, and the growth stage of pigs. **(A)** Change in swine influenza A virus (swIAV) seroprevalence according to the publication year shown by a weighted scatter plot. Solid red line denotes the regression model involved with the seroprevalence proportion, the small sample size, and the publication year. Solid blue line indicates regression describing the association among the proportion, the large sample size, and the publication year. The dashed lines indicate 95% CIs of the regression model. **(B)** swIAV seroprevalence by each growth stage using the bar plot. Circles or triangles indicate individual studies and the size of the figures corresponds to the weighted average of effect size of each study. The sample size is represented by red (small) and blue (large) colors. Error bar represents 95% CIs.

Data visualization was also attempted to measure the impact of the growth stage of pigs as a predictor variable that was significantly associated with the variability of the effect size. These variables were not included in the meta-regression analysis because they caused multicollinearity in the prevalence and were not study-level covariates for seroprevalence; thus, they failed to reveal their impact in the linear model. To identify the underlying association, a non-linear relationship was visualized between the growth stage and the covariates, publication year and sample size, involved in the meta-regression model using weighted scatter plots (Figures 5, 6).

The visualization indicated a temporal decline in swIAV prevalence by placing the publication year on the x-axis (Figure 5A). In this graph, differences in the trends of swIAV prevalence were not observed between studies with a small sample size of <600 (*R*^2^ = 57.5%; estimated coefficient of slope = −0.25 [−0.40; −0.11]) and those with a large sample size of >600 (*R*^2^ = 58.4%; estimated coefficient of slope = −0.25 [−0.45; −0.05]) (Figure 5A). Four studies depicted by the empty red squares in Figure 5A (37, 38, 43, 44) were published before 2008 and were conducted using a small number of pigs (<600 pigs). Furthermore, these four studies examined pre-finishing pigs, and the overall prevalence was 0.30, which was higher than that of the other growth stages (Figure 5B). Studies conducted since 2008 (39, 47–50, 54, 55, 57, 60–62) have used pigs of various sample sizes in the finishing stage. In these studies, a prevalence of < 0.10 was recorded, which was lower than that of pre-finishing pigs (Figure 5A).

Significant temporal trends in swIAV seroprevalence were not detected with respect to publication year and other covariates (sample size and growth stage) (Figure 6A). The seroprevalence in small-sample studies (<750) increased over time (*R*^2^ = 0.0%; estimated coefficient of slope = 0.08 [−0.12; 0.27]), whereas the seroprevalence in large-sample studies (> 750) decreased over time (*R*^2^ = 0.0%; estimated coefficient of slope = −0.09 [−0.37; 0.19]) (Figure 6A). Regarding the association between seroprevalence and growth stage, a non-linear trend was observed (Figure 6B). A high seroprevalence was observed in sows (=0.71) and farrowing pigs (=0.56) (Figure 6B). In the post-weaning and growing stages, a relatively low seroprevalence was observed (post-weaning stage = 0.26; growing stage = 0.28). The seroprevalence of finishing pigs was 0.49, which was lower than that of sows and farrowing pigs but higher than that of post-weaning pigs. Overall, publication year and sample size did not significantly affect the seroprevalence. The high seroprevalence in sows and farrowing pigs gradually decreased, and then increased in finishing pigs.

#### Publication bias

**Figure 7:**
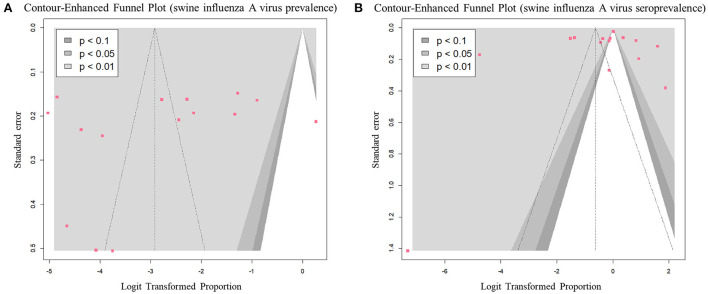
Contour-enhanced funnel plots of eligible studies for each outcome: **(A)** prevalence of swine influenza A virus (swIAV) and **(B)** seroprevalence of swIAV in South Korean domestic pigs.

All studies that reported prevalence, except for one, were distributed in the left significant region (*P* < 0.01) based on a logit value of 0 (Figure 7A). This symmetrical distribution in the funnel plot suggested a low risk of publication bias in the prevalence data (*p*-value of Egger's test = 0.41). The studies reporting seroprevalence were symmetrically distributed between significant (*P* < 0.1) and non-significant regions (*P* > 0.1), based on a logit value of 0 (Figure 7B). This symmetrical distribution in the funnel plot, with a *p*-value of 0.41 in Egger's test suggested a low risk of publication bias in the seroprevalence data.

### Discussion

This work is the first meta-analysis and systematic review of the prevalence and seroprevalence of swIAV in South Korea, although many epidemiological studies on swIAV have been reported in the region. From the relevant data of 27 articles that met the selection criteria, approximately 5% (448/15,279, 95% CIs: 2–12%) prevalence and 35% (11,516/29,095, 95% CIs: 14–63%) seroprevalence of swIAV in domestic pigs were reported. Research conducted in 17 regions in China from 2016 to 2021 found that domestic pigs had a swIAV seroprevalence of nearly 50%, which was slightly higher than the pooled seroprevalence in South Korea reported in this study (64). A systematic review of swIAV surveillance in southeast Asia showed a prevalence of <6% and seroprevalence between 5 and 25% in domestic pigs (65). Similarly, in this study, the swIAV seroprevalence was observed to have a higher proportion and wider 95% CIs than the swIAV prevalence (Figure 2). Pigs infected with swIAV generally undergo an incubation period of 1–3 days and recover quickly within 5–7 days of the onset of clinical symptoms (1, 66). Conversely, antibodies against swIAV are detected 7 days after infection and are maintained at a high level for 8–12 weeks thereafter (66–68). As the antibody-positive cases included individuals who had recovered from swIAV, the seroprevalence would tend to be relatively high compared to the prevalence.

In the subgroup analysis, to explore potential sources of high heterogeneity among the pooled prevalence and seroprevalence, six significant covariates were revealed (Supplementary Tables 5, 6). Three covariates, i.e., publication type, sample type, and detection method, were not included in the final regression model and visualization due to lack of relatedness. Five of the published studies (37, 43, 44, 49, 54) examined samples obtained from at-risk populations, resulting in a higher swIAV prevalence in published studies compared to that in two other unpublished studies (i.e., dissertations and government reports). The results indicated that publication type did not properly explain the changes in the pooled prevalence. Regarding sample type and detection method, Jun et al. (43) conducted IHC staining with lung tissue obtained from carcasses and reported the highest positive rate among other sample types or detection methods, which caused substantial heterogeneity in the analysis. Except for the report by Jun et al., other studies that detected the virus in the lung tissue showed a similar prevalence rate to studies examining the virus in nasal swabs. The existence of outliers may hinder the interpretation of the sample type and detection methods as significant covariates that could generate the heterogeneity shown in the meta-analysis. Thus, the three variables (publication type, sample type, and detection method) were not included in the final model to prevent a confused prediction.

In this study, the final prediction model was constructed by combining the meta-regression model and the adopted visualization using scatter plots with the screened covariates, publication year, sample size, and growth stage (Figures 5, 6). The prevalence over the years from 2008 continuously decreased, and studies published before 2008 examined pre-finishing pigs and showed a high prevalence rate (Figure 5A). Furthermore, studies conducted with a small sample size (<600) of pre-finishing pigs showed a tendency for high prevalence of swIAV (Figure 5B). Studies examining finishing pigs showed a relatively lower prevalence with narrow 95%CIs than the previous growth stage because of studies with large sample sizes (Figure 5B). A previous publication stated that swIAV isolation rates were highest in pre-finishing pigs before 10 weeks of age (69). Viral infection persists owing to the presence of susceptible pre-finishing stage pigs with reduced maternal immunity levels (1). In South Korea, pig housing facilities are generally classified according to their growth stages (70). Although this separation of facilities with growth stages provided an appropriate environment and care for each growth stage, diseases that are common during specific growth stages could continue to circulate in the facility.

In the visualization of the pooled seroprevalence data, trends were identified between growth stage and seroprevalence (Figure 6B). A high seroprevalence was observed in sows, which suggested that swIAV is actively circulated among sows (Figure 6B). The swIAV antibodies generated in infected sows are usually transferred to farrowing piglets via colostrum (71), but the levels of maternally derived antibodies decline over a period of 4–14 weeks in farrowing piglets (1, 72), resulting in a gradual decrease in the seroprevalence of the population of post-weaning pigs during the growth phase. Additionally, the visualization indicated that seroprevalence increased again during the finishing stage. Given the high prevalence rate in the pre-finishing stage (Figure 5B), the pigs at this stage may be intensively infected with swIAV and antibodies to the infection will be produced in pigs that moved on to the finishing stage (66–68). In summary, although meta-regression analysis determined that publication year and sample size influenced prevalence and seroprevalence, the visualized results showed that growth stage had a greater effect on prevalence and seroprevalence data than publication year or sample size. The comprehensive approaches that include not only the statistical analysis of data but also the process of visualizing data are critical to fully understand this risky situation. A solid basis for the interpretation of the analysis results could be built by cross-checking factors that are meaningful in statistical analysis (32).

Notwithstanding the fact that meta-analysis is a useful tool to show the pooled prevalence (73), the researchers identified several limitations in this study. First, other relevant covariates, such as a growth environment in pig farms or a seasonal factor to explain the between-study variance of the dependent variable, were not sufficiently reported in the selected primary studies despite the quality of their work. A previous study suggested the possibility of swIAV circulation depending on the breeding environment and density of pigs (9). Swine influenza A viruses is continuously detected throughout the year, with seasonal peaks worldwide (1, 74). Furthermore, the details of the growth stages were not reported in a few of the included studies. Bias could have been introduced due to insufficient details of the environmental characteristics of the included studies. Second, 27 studies including a total of 15,279 samples for prevalence and 29,095 samples for seroprevalence were included in the meta-analysis, which may be considered an insufficient number of studies to draw conclusions. Interpretation needs to be carried out with caution, but this work was processed with a validated appraisal tool, considering that a meta-analysis can be conducted even when there are only two relevant studies (75). Furthermore, meaningful results were obtained by applying appropriate statistical approaches in meta-analysis methods with a small number of studies (76, 77).

In conclusion, this systematic review and meta-analysis demonstrated that the risk of swIAV circulation is high in the South Korean swine industry. In their role of mixing vessels for the influenza virus, the domestic pigs play a key role in the emergence of new types of epidemic zoonoses. Scientific evidence provided by this analysis and the relevant influencing factors on the pooled data may be practically adopted for future epidemiological investigations in the region, which is necessary to relieve the risk of swIAV. More rigorous cross-sectional studies must be carried out in the future to build adequate preventive policies.

## Data availability statement

The original contributions presented in the study are included in the article/Supplementary material, further inquiries can be directed to the corresponding author.

## Author contributions

SL: conceptualization, methodology, data extraction, data curation, quality assessment, methodology, data analysis, visualization, writing—original draft preparation, and writing—review and editing. EN: data extraction, data curation, and quality assessment. J-WS: conceptualization, project administration, and writing—review and editing. GW: conceptualization, methodology, project administration, supervision, writing—original draft preparation, and writing—review and editing. All authors contributed to the article and approved the submitted version.

## Funding

This subject was supported by the National Institute of Wildlife Disease Control and Prevention as Specialized Graduate School Support Project for Wildlife Disease Specialists.

## Conflict of interest

The authors declare that the research was conducted in the absence of any commercial or financial relationships that could be construed as a potential conflict of interest.

## Publisher's note

All claims expressed in this article are solely those of the authors and do not necessarily represent those of their affiliated organizations, or those of the publisher, the editors and the reviewers. Any product that may be evaluated in this article, or claim that may be made by its manufacturer, is not guaranteed or endorsed by the publisher.
